# Exploring characteristics of the corner sections of a domain wall trap nanostructure with the two-field direction method

**DOI:** 10.1039/c8ra08528e

**Published:** 2018-12-14

**Authors:** Vu Nhut-Minh Ho, Le Duc-Anh Ho, Minh-Tung Tran, Xuan-Huu Cao, Vinh-Ai Dao, Duy-Hien Tong, Duc-The Ngo, Duc-Quang Hoang

**Affiliations:** Applied Computational Civil and Structural Engineering Research Group, Faculty of Civil Engineering, Ton Duc Thang University 19 Nguyen Huu Tho Street, District 7 Ho Chi Minh City 700000 Vietnam hoangducquang@tdtu.edu.vn; Faculty of Applied Sciences, Ton Duc Thang University 19 Nguyen Huu Tho Street, District 7 Ho Chi Minh City 700000 Vietnam; Faculty of Civil Engineering, Ton Duc Thang University 19 Nguyen Huu Tho Street, District 7 Ho Chi Minh City 700000 Vietnam; Advanced Program in Electronics & Communication Engineering, Da Nang University of Science and Technology 54 Nguyen Luong Bang Da Nang 550000 Vietnam; Future Materials & Devices Laboratory, Institute for Fundamental and Applied Sciences, Duy Tan University Ho Chi Minh City 700000 Vietnam; Institute for Computational Science, Ton Duc Thang University 19 Nguyen Huu Tho Street, District 7 Ho Chi Minh City 700000 Vietnam; Electron Microscopy Centre, School of Materials, University of Manchester Oxford Road Manchester M13 9PL UK

## Abstract

A 2D polycrystalline permalloy domain wall trap nanostructure with a thickness of 20 nm was studied. The structure was alternatively designed and patterned using QCAD/L-Edit software and focused-ion beam technique. With this design, a magnetic domain wall can be created and propagated with a sequence of two-field directions in a Lorentz microscopy. The trap consists of two horizontal nanowires and three 90°-tilted ones. Each nanowire has an in-plane dimension of 200 × 1000 nm^2^. The trap corners were curved to allow a created domain wall that easily moves through the structure. A head-to-head domain-wall aims to create using a continuous field, this created wall can be propagated in the trap using a sequence of two-field directions. The designed trap was simulated using the Object Oriented Micro-Magnetic Framework software. Lorentz microscopy and simulation results indicate that the propagation of a domain wall is strongly affected by the precise roughness behavior of the trap elements. Domain wall pinning and transformation of wall chirality are sensitively correlated to the corner sections of the trap structure and field directions at a certain regime. Using the two-field direction method enables us to explore characteristics of the corner sections of the patterned trap nanostructure. This study is vital to fabricate an optimal nano-trap which supports a reproducible domain wall motion. This also suggests a useful method for the domain wall propagation using sequences of two-field directions. This work provides a better understanding of wall creation and propagation in polycrystalline permalloy curved nanowires which are of interest for concepts of nonvolatile data storage devices.

## Abstract 2

A domain wall trap structure which supports a reproducible wall motion using the two field direction method with variations of the two field orientation angles (±*θ*).

## Graphical abstract

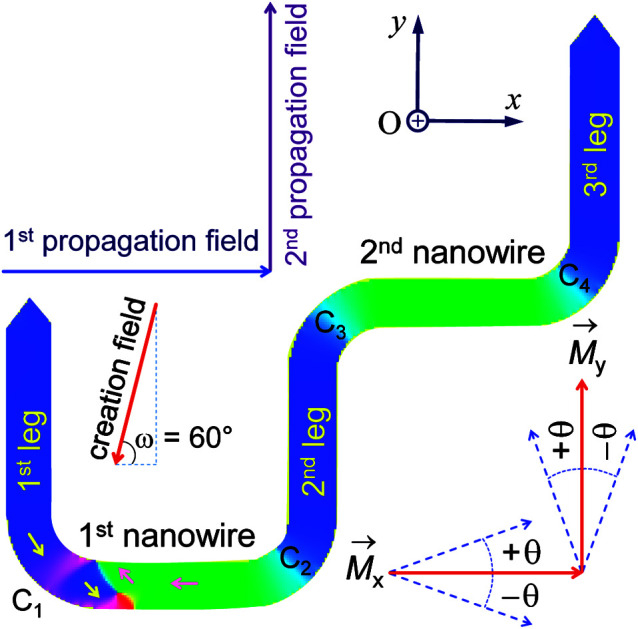

## 1 Introduction

Propagation of a magnetic domain wall (DW) in ferromagnetic nanostructures has attracted much attention in recent years.^1–8^ The concepts of DW propagation are mainly integrated in various potential applications, *i.e.* magnetic logic gates and memory devices.^1,3,7,8^ A number of parameters which directly affect the DW propagation/stability in such applications, *i.e.* temperature, structural dimension/geometry, DW propagation methods, wall types/chiralities.^2,4–12^ These parameters can be engineered to either allow or pin DW movements in those structures.^2,4–7,9–14^ To fabricate such devices for the real life applications, a further understanding of relative parameters that link to characteristics of DW motion is really important. As mentioned in our previous work and by other authors, a domain wall trap (DWT) structure and its characteristics were mainly studied.^2,4,13–15^

**Fig. 1:**
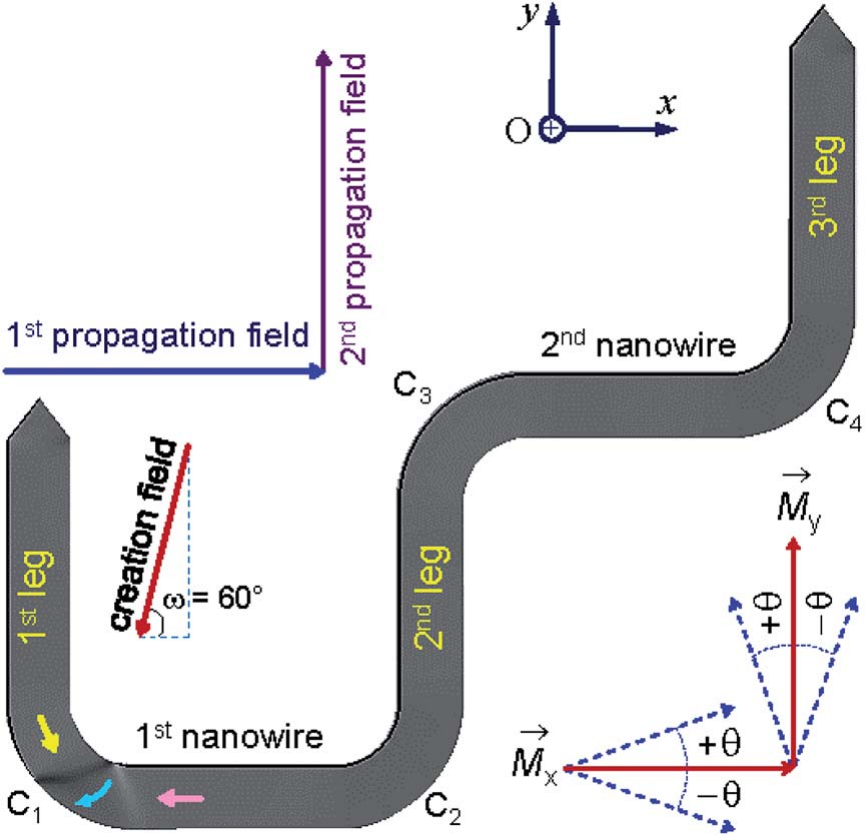
A domain wall trap (DWT) structure which consists of two horizontal nanowires and three other-90°-tilted ones, it was simulated using the OOMMF software.^16^ Each nanowire has an in-plane dimension of 200 × 1000 nm^2^ and a thickness of 20 nm. This trap structure allows to create a single head-to-head DW (H2H-DW), and the created DW can propagate from the first nanowire to the second one using the two field direction method.^14^ Results of which are discussed in the text.

Among a number of DWT-like nanostructures which were investigated, a new geometry has been created in our previous work.^14^ With this design, a single DW can be created at a certain location in the given structure, *i.e.* corners and/or nanowires, the created DW is then propagated in the structure using a two-field direction method.^14^ The composed geometry consists of two horizontal nanowires and three other 90°-tilted ones. Each nanowire has an in-plane dimension of 200 × 1000 nm^2^. This structure partly proved that it has an ability to support a head-to-head (H2H) DW that could be reproducibly moved in the alternative 90°-switching of two-field directions, represented as a combination of blue and violet arrows in Fig. 1. Using this propagation method, the structure required small fields to propagate a created transverse DW (TDW) through the structure, *i.e.* 250 Oe (simulation) or from 12 Oe to 25 Oe (experiment).^14^ Despite the trap thickness of 20 nm,^2^ TDWs are often found in the simulated structure using the Object Orientated Micro-Magnetic Framework (OOMMF) software,^16^ whilst vortex DWs (VDWs) are more often appeared in the patterned structure. Hence, this work aims to discuss on experimental results observed from the given structure using the two-field direction method with a variation of field angle (±*θ*) around the two in-plane magnetization components (*M*_x_ and *M*_y_), also indicated in the inset of Fig. 1. The structure was directly fabricated and characterized using focused-ion-beam (FIB) and Lorentz transmission electron microscopy (LTEM) techniques, respectively.^14,17,18^ Prior to each DW propagation, a continuous biased-field or creation field was applied with an unchanged angle of *ω* = 60° to the two horizontal nanowires, as shown in the inset of Fig. 1. This procedure aims to create a DW at the first corner of the structure (C_1_). A question remained from this structure is, why de-pinning fields (*H*_depin_) required at the trap corners are different, particularly in the patterned structure. In other words, the potential energy landscapes at those trap corners can be experimentally explored with the two field direction method, results of which will be discussed in the following sections.

## 2 Structural designs and simulations

A number of experimental methods have been used to create DWs in various nanostructures with different geometries.^19–25^ Magnetic fields or electric currents with either continuities or pulses, were usually used to create/propagate DWs in such structures with/without protrusions.^26–28^ One of those methods is, an injection pad was attached to one end of a nanowire. Using such injection pads, a single DW was successfully created. The nucleated DW however does not propagate uniformly due to the edge roughness behaviour of those structures. Such characteristics come from those restricted geometries which act as potential barriers/wells, a combination effect of those energy landscapes resists/allows the domain wall propagation to a certain degree. Some few structures with restricted dimension geometries were modified and investigated in our previous work.^14^ Among those structures, a single DW has created and propagated quasi-uniformly through the DWT structure, as given in Fig. 1. To gain a better understanding of a correlation between structural properties and propagation field directions, the DWT structure was designed and patterned using the QCAD/L-EDIT software and FIB technique, respectively.^14^ This tilting field method is available in the modified field emission gun LTEM (FEG LTEM-CM20).^18,29,30^ This technique might provide a DW propagation field range for each DWT corner, *i.e.* particularly at the trap curvatures linking the horizontal nanowires and the vertical ones, denoted as the 1st and 2nd nanowires, and the 1st, 2nd and 3rd legs, respectively. Domain wall pinning, de-pinning and transformation of wall chirality are also characterized.

**Fig. 2:**
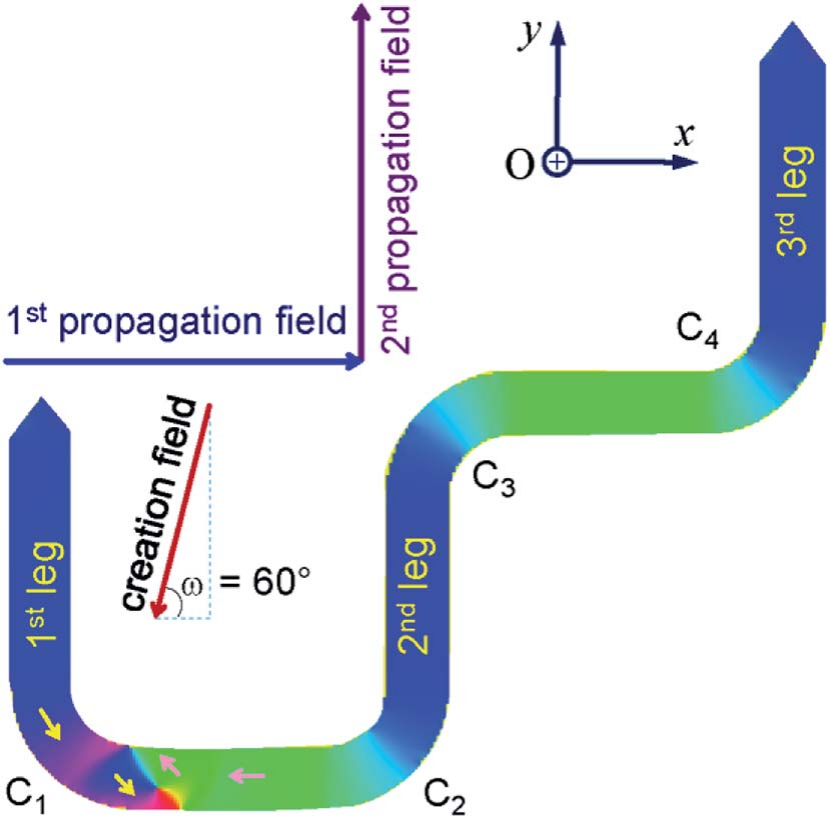
An RGB colour image of the DWT structure which has a H2H vortex DW (H2H-VDW) created at the first corner of the trap structure (C_1_).

Fig. 1 and 2 show that a T/VDW can be created at the C_1_ corner of the DWT structure even if the same creation field is applied with an angle of *ω* = 60°. Formation mechanisms of these wall types are sensitively dependent on the initial stages of the OOMMF simulations. Such walls can also be created/propagated using the Lorentz microscopy.^18,21–25^ Those simulation results were observed using the OOMMF software where magnetizations in either side of the created DW were defined with different colours, *i.e.* the magnetization vectors pointing in the left- and right-directions were defined as black and red colours, respectively.^13^ Herein, the OOMMF simulation results are based on the Landau–Lifshitz–Gilbert equations for the precession and damping of magnetization under an external magnetic field.^2,16,31–33^ The precession dynamics of the magnetization vector (***M***) around an external field (***H***_e_) can be expressed as,1
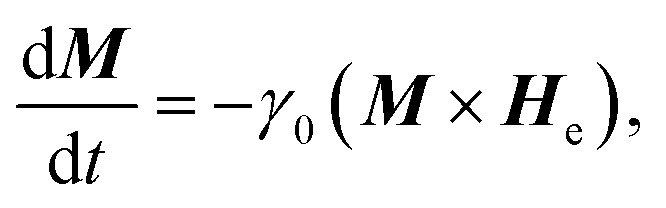
where, d***M***/d*t* is the time derivative of the magnetization, *γ*_0_ is the gyromagnetic ratio, and ***H***_e_ is an effective field that relates to the total energy, *E*, of a ferromagnetic system with a volume (*V*), described by the following equation,2
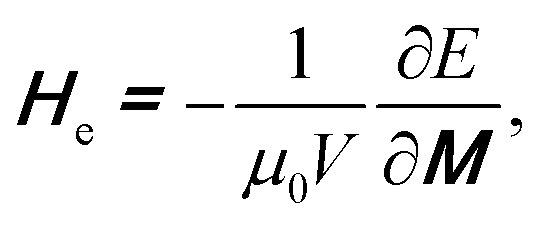


The above relationship describes a continuous precession, however it does not account for a dissipation in energy. Such loss diminishes the precession of ***M*** under ***H***_e_. A dissipation term is thus added into (1), and obtained,3
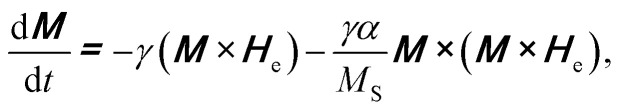
where, *λ* is the damping parameter, *M*_S_ is the saturation magnetization, and *α* is the damping coefficient (= *λ*/*γM*_S_). The first term of eqn (3), – (***M*** × ***H***_e_), describes the precession of ***M*** around ***H***_e_, while the second term, – ***M*** × (***M*** × ***H***_e_), refers to the dissipation of energy and, relates to the motion of ***M*** towards ***H***_e_.

During a simulation, the equation is re-evaluated for each spin–spin interaction until the system reaches an equilibrium state, *i.e.* stable or metastable state. Those states might achieve after a number of iterations or simulation time. Therefore, a minimum value of the torque, d***M***/d*t*, was typically chosen, *e.g.* 10^−5^ A (m s)^−1^. The process of finding the equilibrium state is re-evaluated at each step of increasing the applied field. For the permalloy sample, the OOMMF simulation can ideally include all atoms of the system. Computing power and time are however limited, the process of finding the equilibrium is therefore unachievable. Hence, the principle of OOMMF operation is that the magnetic system is divided into a set of discrete three-dimensional cells. Each cell of the mesh is considered as a spin unit with a magnetic moment, *m*_i_. The cell-size is an important parameter which strongly affects the outcome of a simulation, it is usually chosen close to the characteristic exchange length of the permalloy in order to obtain realistic results.^33–35^ All simulations of this work used a cell-size of 5 nm, this size is comparable to the exchange length, *l*^Py^_ex_ = (2*A*/*μ*_0_*M*_S_^2^)^1/2^ = 5.3 nm, *A* is defined as the exchange stiffness constant and is temperature dependent.^36^ The parameters used for the standard permalloy are, *M*_S_ = 8.6 × 10^5^ A m^−1^ and *A* = 1.3 × 10^−11^ J m^−1^. The damping parameter (*α*) becomes important when the magnetization dynamics is evaluated, *α* can be used between 0.01 and 0.3 for permalloy.^33,37^ However, properties of the trap can be modified after the deposition and patterning processes,^38–40^ a higher *α* value was therefore used, *α* = 0.5.

Each OOMMF simulation output data file can be separated into three magnetization components, *M*_x,y,z_. MATLAB software was used to calculate from OOMMF images directly. The image formation in MATLAB code is calculated using the following equation,^41^4
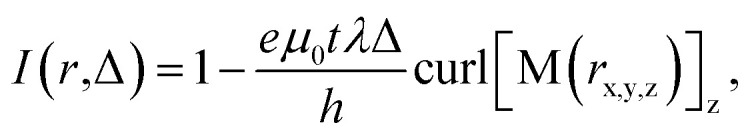


The above relation of the three magnetization components in the sample and microscopy parameters will be used to interpret the Lorentz image intensity of the DWT structure.^18,41,42^ This was calculated for the linear regime of the TEM transfer function in Fourier space with de-focused value (Δ, defocus is the aberration where an image is out of focus) in the range of 0 and 160 nm, 0 ≤ Δ ≤ 160 nm.^41^ Nevertheless, experimental results are largely concerned with the non-linear regime of Δ, *e.g.* Δ = 3600 μm, this aims to improve the image contrast of Fresnel images. As assumed that the *M*_z_ does not contribute to the Lorentz imaging contrast. The *M*_x_ and *M*_y_ components are mainly contributed to the Lorentz image intensity profile which is proportional to curl(*M*)_z_.^13,41^

## 3 Experimental details

### 3.1 FIB patterning the DWT structure

The designed DWT structure was experimentally patterned using a Ga^+^ FIB irradiation method. The FIB used herein was an FEI × T NOVA NanoLab 200 DualFIB.^13,14,38–40^ The FIB patterning can be separated into two main steps: (1) a continuous 20 nm-thick Py film was evaporated onto an electron transparent Si_3_N_4_ TEM membrane using a thermal evaporator with the evaporation rate of 0.03 nm s^−1^.^13,14,38^ The TEM membrane consists of a 35 nm-thick amorphous Si_3_N_4_ supported on a 500 μm-thick silicon frame with a 100 × 100 nm^2^ electron transparent window, obtained from TED PELLA, INC.^43^ The evaporated Py film thickness was controlled and monitored using a quartz crystal microbalance technique where a correlation between mechanical oscillation and resonant frequency was detected. The use of Si_3_N_4_ TEM membrane allows the patterned structure which is directly characterized using the Lorentz microscope.^18^ (2) The FIB irradiation technique for the DWT structure patterning is fully available in the Kelvin nanocharacterization centre at the University of Glasgow. With this method, the patterned DWT structure was simply isolated from the continuous 20 nm-thick evaporated Py film,^40,44^ as described in Fig. 4.

In the FIB patterning process, each pixel in the irradiated area can receive the same dose,^17^ a 0% ion-beam overlap is often used for patterning, Fig. 4(a). The ion-beam overlap condition can be varied between 0% and 50% to obtain a smoother edge roughness. Prior to FIB irradiations, the milling area was redefined using the edge-stream program. This aims to trace out the edge profile of the patterning structure with vector scanning strategy. Such jobs allow the milling area around the patterned DWT structure process with a single/multiple cut. Depending on the size of milling area which will be removed, various input-parameters for the FIB patterning are defined accordingly, *i.e.* FIB working screen location, dwell time, ion-beam current, pixel overlap/size, sputter rate, milling depth. The Ga^+^ ion beam current of 9.7 pA was used, this is equivalent to the ion-beam diameter of 10 nm. A longer total dwell time with multiple passes often uses to obtain smooth edge profiles rather than using a single pass with a long dwell time.^39,44,45^ Depending on each research direction, patterning conditions can be used differently to obtain expected results.

A number of effects have been investigated on thin ferromagnetic films using FIB irradiation.^17,39,40,44^ However, we simply used the FIB technique with the Ga^+^ ion beam to isolate the designed DWT structure from the continuous Py film, and to obtain a higher edge-profile quality with the 50% ion-beam overlapping. We used the patterning time for the first cut was around a minute to avoid beam-drift issues. As an example, a FIB-SEM image of the patterned DWT structure is given in Fig. 4(b). Based on the FIB-SEM image contrast, effects of re-deposition can be visualized at the along edges of the patterned structure. Magnetic properties of the trap was characterized with the Fresnel imaging mode of the FEG-LTEM Philips CM20 microscope,^13–15,18,22–25^ Δ = 3600 μm was used for all measurements. To reduce charging effects during the FIB irradiation and TEM imaging acquisition, a very thin conducting gold layer of 5 nm was deposited to the backside of Si_3_N_4_ TEM membrane using another sputtering deposition technique with a 99.99% purity-gold target.^22,23^

### 3.2 Magnetizing experiment

The magnetic properties of the patterned DWT structure were characterized using the Philips CM20 FEG-LTEM/STEM. The technique has been modified for advanced magnetic imaging purposes.^18,41,45,46^ With this technique, the sample is situated between the upper and lower pole-pieces of the objective lens. This means that the specimen is immersed in a magnetic field strength of ≈20 000 Oe, this field aligns parallel to the optical axis of the microscope. Such field strength is sufficient to destroy the magnetic state of most samples. The objective lens of the microscope is usually switched off. Two additional mini-lenses are therefore used to replace functions of the objective lens, and those lenses create a field-free environment for the imaging modes of magnetic samples in both Fresnel and differential phase contrast modes.^18^

**Fig. 3:**
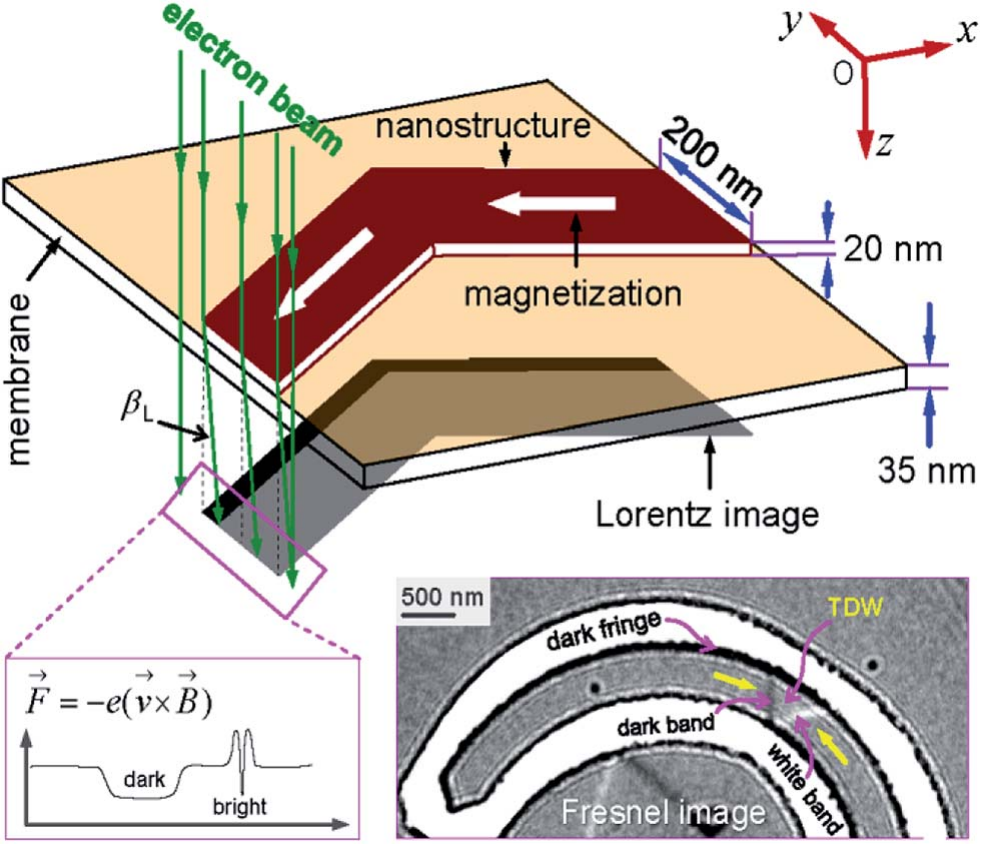
A simplified diagram of the electrons deflected by the classical Lorentz force, *F* = − *e*(*v* × B), where the intensity profile of transmitted electrons at a plane below the specimen is depicted. Darker and brighter bands are shown in the bottom-left corner. Such characteristics will be used to interpret experimental Fresnel images of the Py DWT structure. The imaging contrast arises from the magnetic ripple background appears as a grey band with dark and white boundaries on either side, as seen in the bottom-right corner.

The electrons extracted from the FEG source of a Lorentz microscope pass through a thin magnetic foil. The emergent electrons are deflected by the Lorentz force which produced by the magnetic field within and surrounding the Py nanostructure, as described in Fig. 3. Therein, if *t* and *B*_S_ (*B*_*S*_ = *μ*_0_*M*_S_) are alternatively the specimen thickness and the saturation induction of the magnetic material, the deflection angle (*β*_L_) can be expressed as, *β*_L_ = (*eB*_S_λ*t*/*h*), where *e* = − 1.602 × 10^−19^*C* is the electronic charge, *h* = 6.626 × 10^−34^ J s is the Planck constant and *λ* = 2.51 pm is the electron wavelength in the case of accelerating voltage, *V*_FEG_ = 200 keV.^18^ The Lorentz lens is de-focused by values of ± Δ in the Fresnel imaging mode, either under-focused (+Δ) or over-focused (−Δ) in respect of the focal plane, resulting magnetic contrast arises in the Lorentz image, as seen in Fig. 3. This hints that if the deflected electrons are in-focused, no magnetic contrast in the Fresnel image exists. As shown in the inset of Fig. 3, when an electron beam is transmitted through a thin ferromagnetic Py structure which consists of a 180°-domain wall.^18^ The transmitted electron beam is deflected by the Lorentz force with *β*_L_, this force deflects the electrons from neighbouring domains in opposite directions, *i.e.* both sides of a created TDW. The domain wall appears as bright and dark bands against the neutral grey-background, as seen in the bottom-right corner of Fig. 3. Dark fringes appear along the DWT edges which due to the transition between magnetic and non-magnetic materials.^18^

**Fig. 4:**
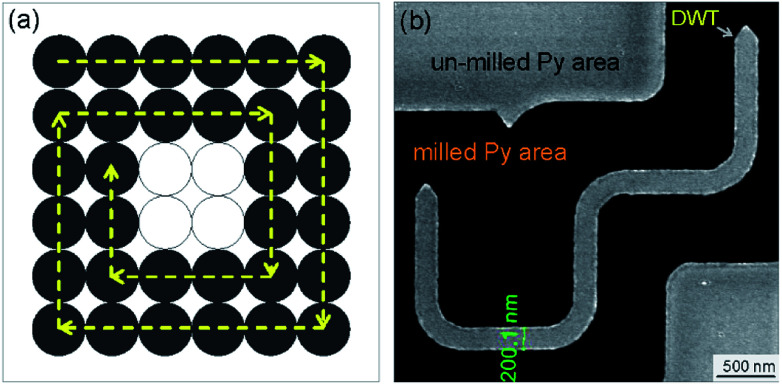
(a) A simplified schematic drawing of the FIB irradiation method which describes the moving path of a 10 nm-diameter ion-beam during a FIB patterning process using the edge-stream program. Those ion-beam spots are indicated by a series of grey-solid-circles. We assumed that the ion-beam spots irradiated on the continuous Py film that have no overlaps (0% ion-beam overlapping). (b) A FIB-SEM image of the Py DWT structure which was patterned by the FIB irradiation with 50% beam overlapping.^17,39,40,44,45^

**Fig. 5:**
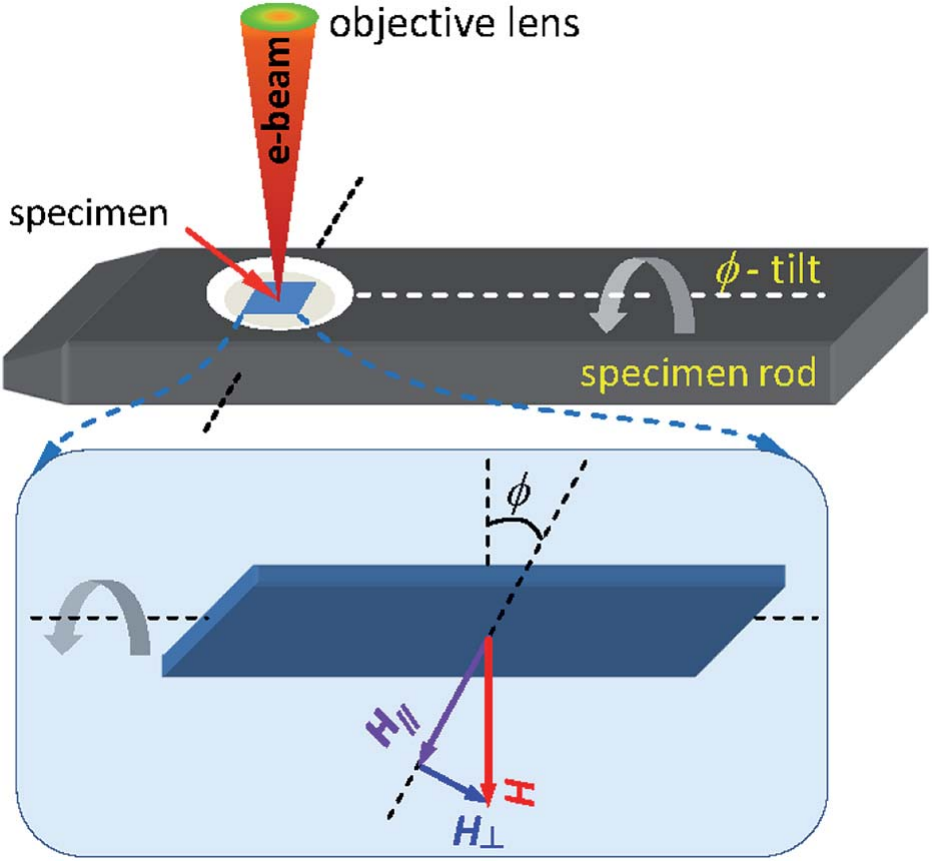
A Si_3_N_4_ membrane mounted in the TEM sample rod where the patterned DWT structure can be magnetized by a DC field (*H*) when the objective/twin lens is on, as given in the upper panel. Parallel and perpendicular field components (*H*_‖_ and *H*_⊥_) applied in respect of the sample surface are also represented in the lower panel.

A simplified schematic drawing of the *in situ* Lorentz TEM measurement using a continuous field,^18^ at which a thin specimen is mounted in the TEM sample rod, as shown in Fig. 5. The rod can be tilted an angle (*ϕ*) to introduce parallel and perpendicular field components, *H*_‖_ and *H*_⊥_. The sample is magnetized by applying a DC field (*H*) produced by the mini-lenses/twin lens, and the field strength can be controlled by adjusting the electric current injected to the objective lens coils. The sample plane is usually oriented to the horizontal plane, the 0°-tilted stage. This means that the objective lens field is perpendicular to the sample plane, no field applies to the horizontal plane. When the specimen plane is titled with *ϕ*, the field component in the horizontal plane, *H*_‖_, can be expressed as, *H*_‖_ = *H* sin(*ϕ*). The maximum field value can be achieved at the 90°-tilted stage. The maximum magnetic field can be produced by the twin lens of the Glasgow LTEM is of 7000 Oe.

## 4 Results and discussion

**Fig. 6:**
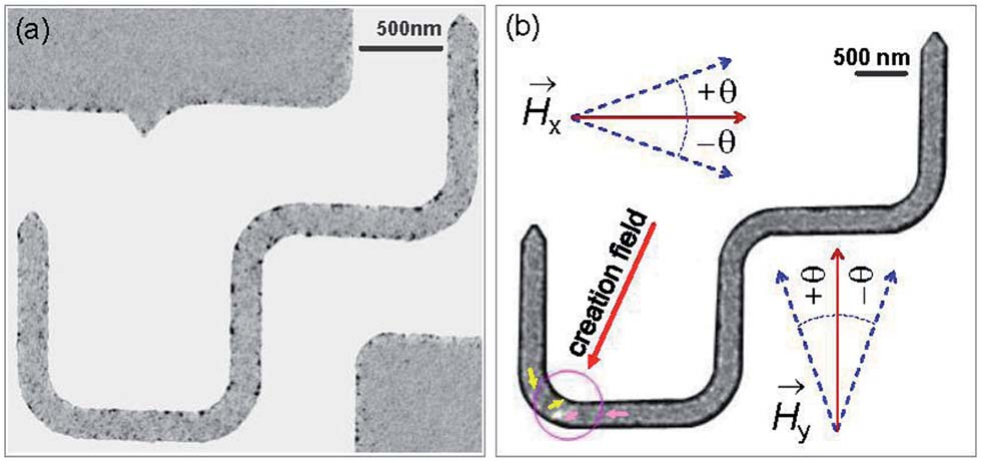
(a) A BF-TEM image of the patterned DWT structure with a thickness of 20 nm. (b) A Fresnel image of the trap where a VDW was created with the creation field of 7000 Oe, indicated by a red arrow and described in Fig. 1 and 2. The created VDW was pinned in the first corner (C_1_) of the structure, similar as a particle or an entity confined to a potential well which is created by the C_1_ corner geometry. To propagate the pinned VDW through the structure, this VDW should be de-pinned out of the corner by a propagation field, the so-called de-pinning field, *H*_depin_, as discussed in the text.

A bright field TEM (BF-TEM) image of the DWT structure patterned by the FIB irradiation method is given in Fig. 6(a), at which the width of nanowires was measured around (200 ± 5) nm. This value is comparable to the simulated one. As also seen from the BF-TEM image, some grains denoted as darker and brighter particles appear along the DWT edges. This indicates that the patterned DWT structure was affected by the irradiation processes of the FIB fabrication. Such imperfect behaviour of the edges induces DW propagation through the DWT structure under the two field-direction method.^13,14^ De pinning field strength (*H*_depin_) at each corner of the DWT structure is particularly emphasized. The constant procedure used herein is, the unchanged field of 7000 Oe was applied about the angle of *ω* = 60° with respect to the easy-axes of the two horizontal nanowires, as described in Fig. 1 and 2, at which either TDW or VDW can be created in the simulated structure, whilst a VDW was experimentally nucleated at the C_1_ area, as seen in Fig. 6(b). The chirality of created VDW sensitively depends on the field strength/angle applied to the structure and structural properties of the C_1_ corner, such effects were partially investigated by other authors.^15,22^

**Fig. 7:**
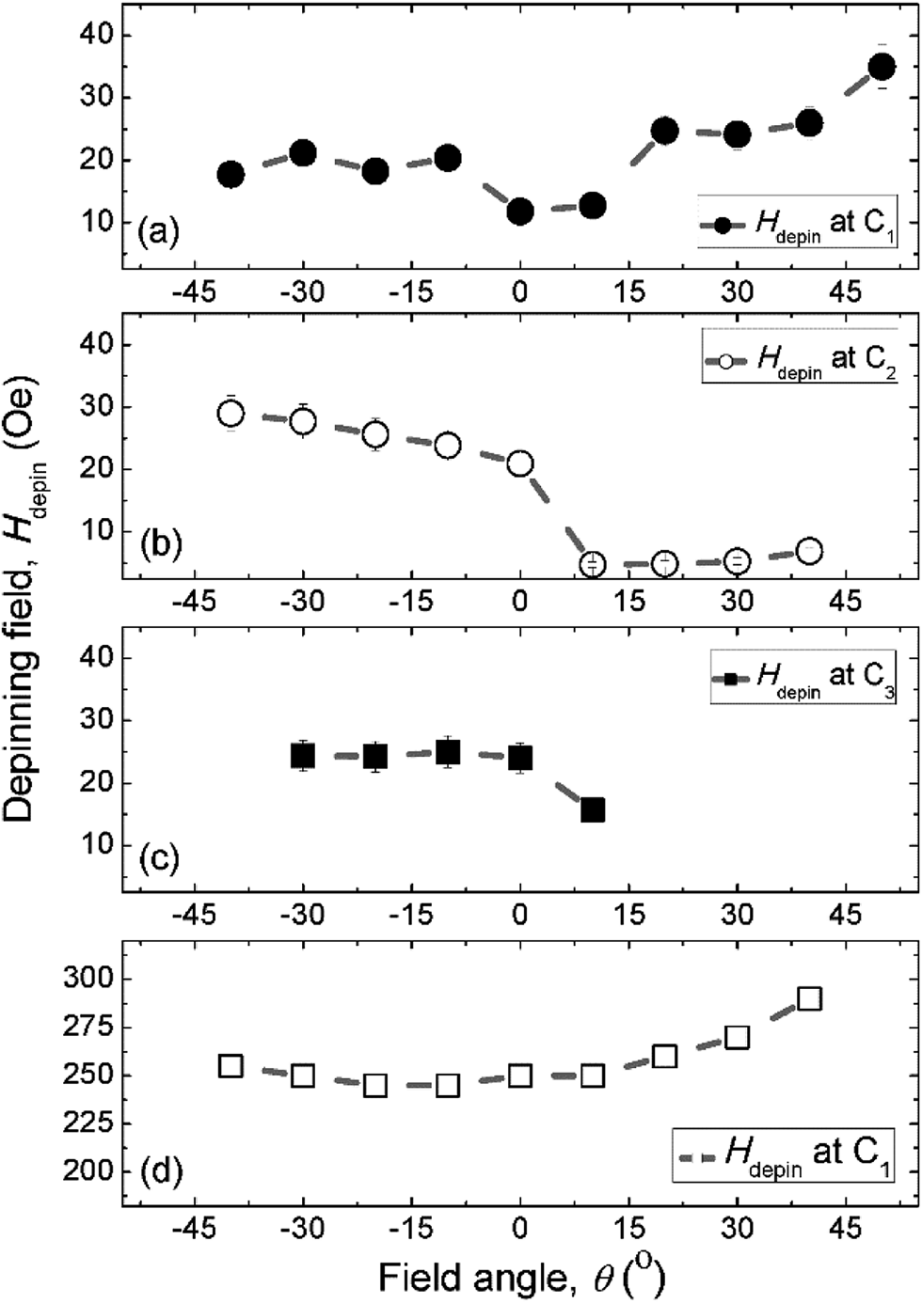
De-pinning fields (*H*_depin_) of the created clockwise-VDWs (CW-VDWs) as a function of the field angles (±*θ*) at the C_1_ (a), C_2_ (b) and C_3_ (c) corners of the patterned structure, while (d) represents a *H*_depin_–*θ* curve for the TDWs created at the C_1_ corner of the simulated one.

We discussed a part of our observations with both simulation and experimental results using a sequence of two field directions (*H*_x_ and *H*_y_).^14^ Using the combination of those field components, a created VDW was successfully propagated from the first to second nanowire. The de-pinning field required to propagate a DW through the DWT corners is more reproducible in the simulation results with the 0°-tilted field, while it is less in the patterned structure. To understand characteristics of DW movements under the two-field direction method with a variation in propagation field directions (±*θ*) at each DWT corner, as illustrated in the inset of Fig. 6(b). This hints that not only the two-field directions apply parallel to the horizontal nanowires and the vertical ones, however the field direction at each sequence also varies, *i.e.* in a range of ±50°. Such experimental procedures can indirectly explore the role of each DWT corner at which the four corners of the patterned structure are considered as four pinning points. De-pinning fields of those corners (*H*_depin_) as a function of field angles/directions (*θ*) in the forward process are plotted for the C_1_, C_2_ and C_3_ corners, as given in Fig. 7(a–c). Such relationship was also simulated using the OOMMF software for the C_1_ corner of the designed DWT structure, as shown in Fig. 7(d), for a comparison.

Fig. 7(a–c) show the experimental results of de-pinning field values (*H*_depin_) as a function of field angles (±*θ*) where the de-pining fields at a certain corner relate to the energies which need to push a created/propagated DW out of the C_1→3_ corners. As seen in the relations between *H*_depin_ and ±*θ* (*H*_depin_–*θ*) that are entirely asymmetric on both sides of the 0°-field angle. This asymmetric behaviour might belong to the dependence of de-pinning fields on a combination effect of the energy landscape at each corner and the local spin configuration inside the created DW in respect of the field direction and/or the VDW chirality.^21,22^ However, the external fields required to de-pin the created/propagated TDWs in the simulated structure at the C_1_ corner in the negative field directions (−*θ*) are slightly lower than that obtained in the positive ones (+*θ*). As discussed, the ±*θ* defined as the field directions oriented to the left- and right-sides of the horizontal and vertical nanowires, as already indicated in Fig. 6(b). The discrepancies of those de-pinning field values on both sides of the parallel field direction (the 0°-tilted field) might originate from the spin configuration of the created/propagated DW and the precise location of the DW at each corner.

The trend of experimental *H*_depin_–*θ* data points, Fig. 7(a), is consistent with the simulated ones, Fig. 7(d). A small difference in those curves at the negative angle (−*θ*) might come from the initial stages of those DWs. The wall created at the C_1_ corner of the patterned structure is a CW-VDW, Fig. 6(b), whilst it is a TDW in the simulated one. Besides, the domain wall energy landscapes at the C_1_ corner are different between the simulated and patterned structures where effects of the edge roughness are incomparable. The de-pinning fields of those created walls at the C_1_ corner with *θ* = 0° are also different, as compared the simulated values to the experimental ones. These variations might also come from thermal effects that were excluded from the simulation, while the experiment was realized at room temperature. Moreover, other parameters could be included in a simulation, *i.e.* pixelation, re-deposition, structural surface, residual field in the Lorentz TEM, quality of the deposited Py film.

When the created DW moves to the C_2_ corner, it is then propagated to the C_3_ under the second field direction (*H*_y_), the de-pinning fields as a function of field angles (*θ*) are given in Fig. 7(b). The *H*_depin_–*θ* curve shows a new trend which differs to that obtained from Fig. 7(a). This hints that the de-pinning field strongly relates to the local spin configuration at an individual area and the DW chirality changed on reaching the C_3_ corner.^14^ The propagated DW at the C_3_ continuously moves to the C_4_ corner with the first field direction (*H*_x_), Fig. 7(c). A difference in those cases, Fig. 7(a and b), is that the propagated DW at the C_3_ area was unstable when the first field direction applied with *θ* > +10°, a couple of data points are therefore excluded from Fig. 7(c).

Based on the discussed results, using a sequence of the two field directions (*H*_x_ + *H*_y_) with different field angles (±*θ*), a created DW successfully propagates from one end of the first nanowire to another end of the second one. Each data point of Fig. 7(a–c), was calculated from five different measurements with the same condition, *i.e. ω*, *θ*, the creation field strength of 7000 Oe. The potential energy landscapes/profiles of those corners/curvatures were indirectly explored with various characteristics, *i.e.*
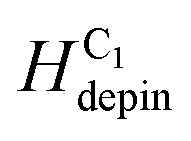
 = 11.8 Oe, 
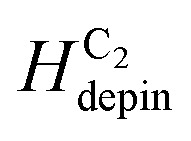
 = 21 Oe, 
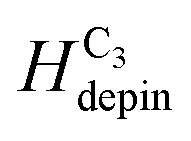
 = 24 Oe. These experiment values belong to the created CW-VDWs propagated with the 0°-field, whilst the simulation value of 
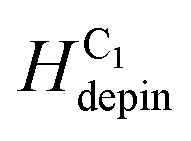
 = 250 Oe belongs to the created TWD. Moreover, the VDW chirality was also changed during the propagation process.^14,22,27^ Such changes relate to magneto-static effects, this leads to DW distortion, results in reducing the total energy of the Py system. This is similar to each created/propagated DW which can be confined to potential wells where a combination effect of shape anisotropies and magneto-static energies at those patterned DWT corners. The characteristics of each DWT corner, for examples, edge roughness, potential energy landscapes, domain wall spin configurations, were indirectly explored using the two-field direction method with a variation of ±*θ* in the forward process. In principle, the DW propagated to the C_4_ corner which could be driven back to the C_1_*via* the reversal process of the two field direction method. However, the DW propagation in the reversal process is less reproducible than that obtained in the forward one. Such discrepancies might result from the propagated DW positions in the forward and reversal processes are somehow different. Moreover, the geometrical parameter of the trap is changed in respect of the field directions in the forward and reversal processes.

## 5 Conclusions

The DWT structure consists of five Py nanowires which was patterned using the FIB irradiation technique. Propagation behaviour of a DW created in the DWT structure was characterized with the Lorentz microscopy and its associated techniques. Moreover, with the switching of 90°-two-field directions, DW propagation in the designed DWT structure was systematically studied by means of the OOMMF simulation. Propagation characteristics of the created TDW in the simulated structure are reproducible with both forward and reversal processes using the 0°-tilted propagation fields. Characteristics of DW movements at each corner of the patterned structure were particularly characterized with different field angles/orientations (±*θ*), as compared to the horizontal plane. A combination of those field directions was mainly investigated, we assigned that a few dominant parameters affected the DW propagation, *i.e.* edge roughness, potential energy landscapes, domain wall spin configurations, geometrical parameters in respect of field directions.

Based on the experimental observations, we also found that the de-pinning fields required at each corner are different in the patterned DWT structure. Such differences mainly come from the local spin configuration in respect of the applied field direction at a particular location of the structure. This leads us conclude that DW pinning and transformation of wall chirality are sensitively correlated to edge roughness and/or structural geometries at a certain area. This hints that effects of shape characteristics and local spin configurations at the curved sections particularly play a crucial role. Our results contributed to a road map of finding a nanostructure which is suitable for the field driven DW motion between two straight nanowires linked by another one using a sequence of 90°-two-field directions, this is also of interest for concepts of high-tech applications.

## Conflicts of interest

There are no conflicts of interest to declare.

## Supplementary Material
